# Impact of masking policy on healthcare-associated acute respiratory infections in 18 hospitals in Southern Ontario

**DOI:** 10.1017/ash.2026.10423

**Published:** 2026-06-01

**Authors:** Thomas Scheier, Anne Bialachowski, Michael Silverman, Tom Szakacs, Marianita Lampitoc, William Ciccotelli, Medina Saffie, Karim Ali, Zain Chagla, Dominik Mertz, for the SWO-IPAC group

**Affiliations:** 1 Population Health Research Institute, Canada; 2 St Joseph’s Healthcare Hamilton, Canada; 3 Western University Faculty of Science, Canada; 4 Brantford General Hospital, Canada; 5 London Health Sciences Centre, Canada; 6 Waterloo Regional Health Network, Canada; 7 Joseph Brant Hospital, Canada; 8 Niagara Health System, Canada; 9 McMaster University, Canada

## Abstract

Whether continuous masking prevents healthcare-associated acute respiratory infections (HA-ARI) remains uncertain. We demonstrate that discontinuing continuous masking policies in 18 acute care facilities in Ontario was not associated with an increase in the incidence of HA-ARIs in hospitalized patients, or with ARI outbreak activity over two consecutive ARI seasons.

## Background

Healthcare-associated acute respiratory infections (HA-ARI) are associated with significant morbidity and mortality.^1,2^ Using face masks continuously for everyone entering a healthcare facility (often referred to “universal masking”) to reduce spread was widely implemented during the pandemic with some continuing this approach during periods of high levels of circulation of ARIs. Data during the SARS-CoV-2 pandemic has shown a potential reduction in the number of HA-ARI using this approach.^3,4^ However, it is uncertain whether to continue these policies postpandemic with a lower burden of circulating ARIs.^5^ Here, we provide results of a comparison between acute care facilities in south-western Ontario that continued continuous masking policies during the ARI season versus those that no longer required continuous masking two consecutive years postpandemic.

## Methods

A survey was developed to assess the implemented masking policies during two ARI seasons, size of healthcare facilities, and number of SARS-CoV-2, Influenza, and RSV HA-ARI cases among patients, and number of outbreaks. HA was defined as onset of symptom >48 h after hospital admission. Data was collected for the 2023/2024 (year 1) and 2024/2025 (year 2) ARI season.

We grouped facilities according to continuous masking policy changes between year 1 and 2: continued (ie, continuous masking in years 1 and 2), discontinued (ie, continuous masking in year 1 but not in year 2), never (ie, no continuous masking in either year). The total number of HA-ARI cases among patients and outbreaks by site and year was calculated for all respiratory viruses combined, and for SARS-CoV-2 specifically. The difference for each site between year 1 and year 2 was calculated and reported as a percentage change from year 1 (ie. each site served as its own control). Findings are reported by group and are displayed as boxplots using R.^6^ Median and IQR are presented. The Kruskal-Wallis test was performed to compare groups. Analyses were stratified by hospital size (<400 vs >400 beds) in a sensitivity analysis.

The study was considered quality assurance and therefore exempt from formal ethics approval (Hamilton Integrated Research Ethics Board).

## Results

Data was provided by 18 healthcare facilities across south-Western Ontario. Characteristics of sites and continuous masking policies are outlined in Supplement Table S1. Continuous masking policies were discontinued, continued or never implemented in 5, 11 and 2 sites, respectively (Supplement Table S1). Overall, the number of cases (year 1: 1,366, year 2: 1,235) and the number of outbreaks (year 1: 142, year 2: 107) decreased numerically.

### HA-ARI cases

**Figure 1.**
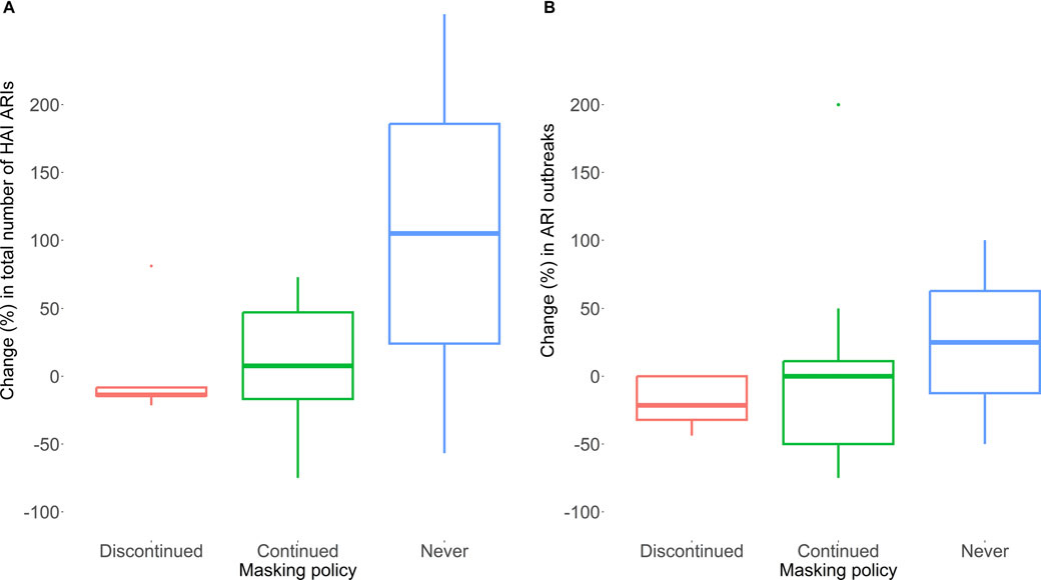
Impact of face masking policies on healthcare-associated acute respiratory infections (HA-ARI). HA-ARI, healthcare-associated acute respiratory infections; (A) Total number of HA-ARIs and (B) number of ARI outbreaks was calculated as difference (in %) for each site between year 1 and year 2. Results were pooled for each policy group and displayed as boxplot (median, lower quartile and upper quartile).

Sites that discontinued continuous masking policies in year 2 had a median decrease in the absolute number of HA-ARI cases of −13.8% (IQR: −14.3%; −8.3%). Sites that continued continuous masking policies in both years had an increase in HA-ARI cases (+7.7% (IQR −16.8%; +47.1%)). Sites without any masking policy in either year also had an increase in ARI cases of + 105.0% (IQR 24.1%; 185.8%) (Figure 1(A)). There was no statistically significant difference across the three groups, though (*p*-value = .89). For SARS-CoV-2 specifically, median changes were −20.0% (IQR −21.5%; −15.1%) for discontinued, −8.1% (IQR −43.3%; +14.5%) for continued, and + 75.4% (IQR 2.0%; 148.8%) for sites with no continuous masking over the two years (Supplement Figure S1A).

### Outbreaks

The number of outbreaks decreased for sites that discontinued masking policies by −21.4% (IQR −32.1%; 0.0%), was stable (0% (IQR −50.0%; 11.1%)) at sites that continued masking policies, and increased at sites that had no continuous masking policies in place over the two study years (+25.0% (IQR −12.5%; +62.5%)) (Figure 1(B)). There was again no statistically significant difference between groups (*p*-value: .96). Sites that discontinued, continued or never had masking policies in place reported a change in the number of SARS-CoV-2 outbreaks between year 1 and 2 of −31.6% (IQR −41.7%; 0.0%), −34.0% (IQR −68.8%; −2.1%) and + 13.0% (IQR −30.4%; +56.5%), respectively (Supplement Figure S1B).

Separating sites by number of beds showed similar results for each of the policy groups with overlapping interquartile ranges for sites that continued versus those that stopped with continuous masking policies (Supplement Figure S2).

## Discussion

Our study suggested that discontinuing continuous masking policies in acute care facilities was not associated with an increase in the incidence of HA-ARIs in hospitalized patients, or with ARI outbreak activity over two consecutive ARI seasons.

The risk of HA-ARIs is driven by a variety of factors such as hospital infrastructure, patient population, existing infection prevention-measures, and the epidemiological situation. In addition, adherence, mask effectiveness and interactions between healthcare workers also impact the spread of ARIs and are often included in modeling studies.^7^ This complex relation between all factors highlights how difficult it is to clearly attribute any protective effect to masking itself, and might explain the conflicting data, even if not exclusively focusing on SARS-CoV-2.^8–10^ The chosen study design, and using each facility as its own control, should minimize the impact of these confounding factors. Epidemiological data showed a similar difference in the incidence in SARS-CoV-2 per 100,000 population for public health units across all policy groups, but a slightly more pronounced increase in influenza cases for units of sites which continued compared to those which discontinued (+58% vs +42%). The total number of cases per 100,000 population for COVID and influenza combined was higher in the first than in the second year. A few more limitations are noteworthy. Firstly, it is difficult to determine compliance with masking policies at all times of day, and the findings must be considered in the context of a real-world experience with imperfect compliance. Second, the number of HAI cases is not normalized per patient-days, but inpatient occupancy consistently exceeds 100% for included sites during the ARI seasons. Third, we focused on masking of healthcare workers and did not separate masking policies for visitors, nor did we investigate if the masking policies of both years were exactly identical at a given site. Given all the limitations, the study cannot rule out a potential small benefit from continuous masking policies.

In conclusion, our findings are reassuring that discontinuing continuous masking during the ARI season was not associated with a sizeable increase in ARI activity. Acute-care health-care facilities might still consider continuous masking in outbreak settings to reduce further spread, or under other extraordinary circumstances as a smaller than detectable relative benefits of continuous masking cannot be ruled out.

## Supporting information

10.1017/ash.2026.10423.sm001 Scheier et al. supplementary material 1 Scheier et al. supplementary material

10.1017/ash.2026.10423.sm002 Scheier et al. supplementary material 2 Scheier et al. supplementary material

10.1017/ash.2026.10423.sm003 Scheier et al. supplementary material 3 Scheier et al. supplementary material

10.1017/ash.2026.10423.sm004 Scheier et al. supplementary material 4 Scheier et al. supplementary material

## Supplementary material

The supplementary material for this article can be found at https://doi.org/10.1017/ash.2026.10423.
