# Supplementary material for: Impact of masking policy on healthcare-associated acute respiratory infections in 18 hospitals in Southern Ontario
Source: Antimicrob Steward Healthc Epidemiol. 2026 Jun 1;6(1):e160. doi: 10.1017/ash.2026.10423 (PMC13227126; doi:10.1017/ash.2026.10423)
Supplement: Scheier et al. supplementary material 1 — Scheier et al. supplementary material [file S2732494X26104239sup001.docx]

**Figure S1:** Impact of face masking policies on healthcare-associated Covid infections (HAI Covid)

A) Total number of HAI Covid and B) number of Covid outbreaks was calculated as difference (in %) for each site between year 1 and year 2. Results were pooled for each policy group and displayed as boxplot (median, lower quartile and upper quartile).

**Figure S2:** Impact of face masking policies on healthcare-associated acute respiratory infections (HA-ARI)

HA-ARI = healthcare-associated acute respiratory infections; A & B) Total number of HA-ARIs and C & D) number of ARI outbreaks was calculated as difference (in %) for each site between year 1 and year 2. Results were pooled for each policy group and displayed as boxplot (median, lower quartile and upper quartile). A and C include only hospitals with >400 beds, B and D include only hospitals with <400 beds.
