# Supplementary figures and images for: Impact of masking policy on healthcare-associated acute respiratory infections in 18 hospitals in Southern Ontario
Source: Antimicrob Steward Healthc Epidemiol. 2026 Jun 1;6(1):e160. doi: 10.1017/ash.2026.10423 (PMC13227126; doi:10.1017/ash.2026.10423)

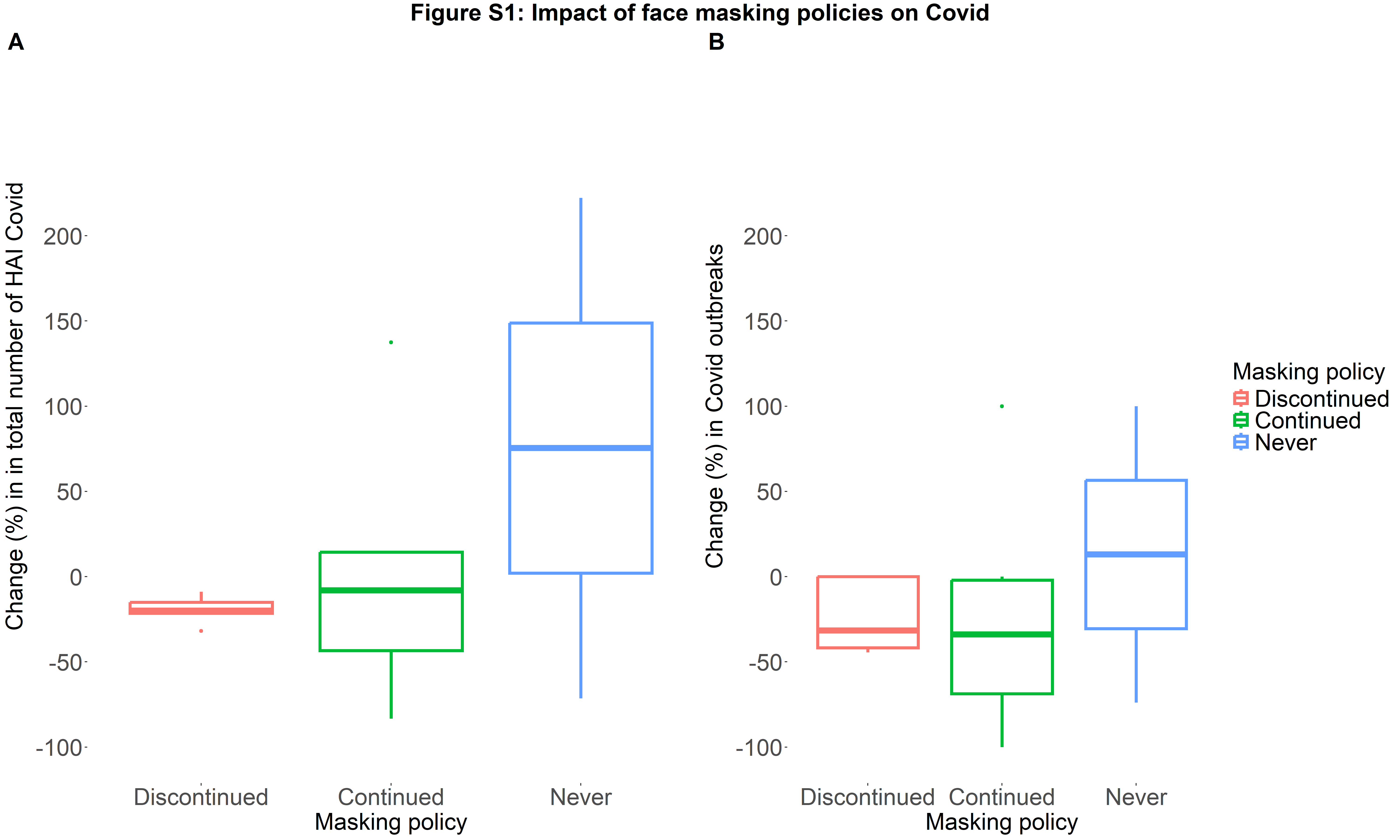

Supplement: Scheier et al. supplementary material 2 — Scheier et al. supplementary material [file S2732494X26104239sup002.zip › FigureS1.png]

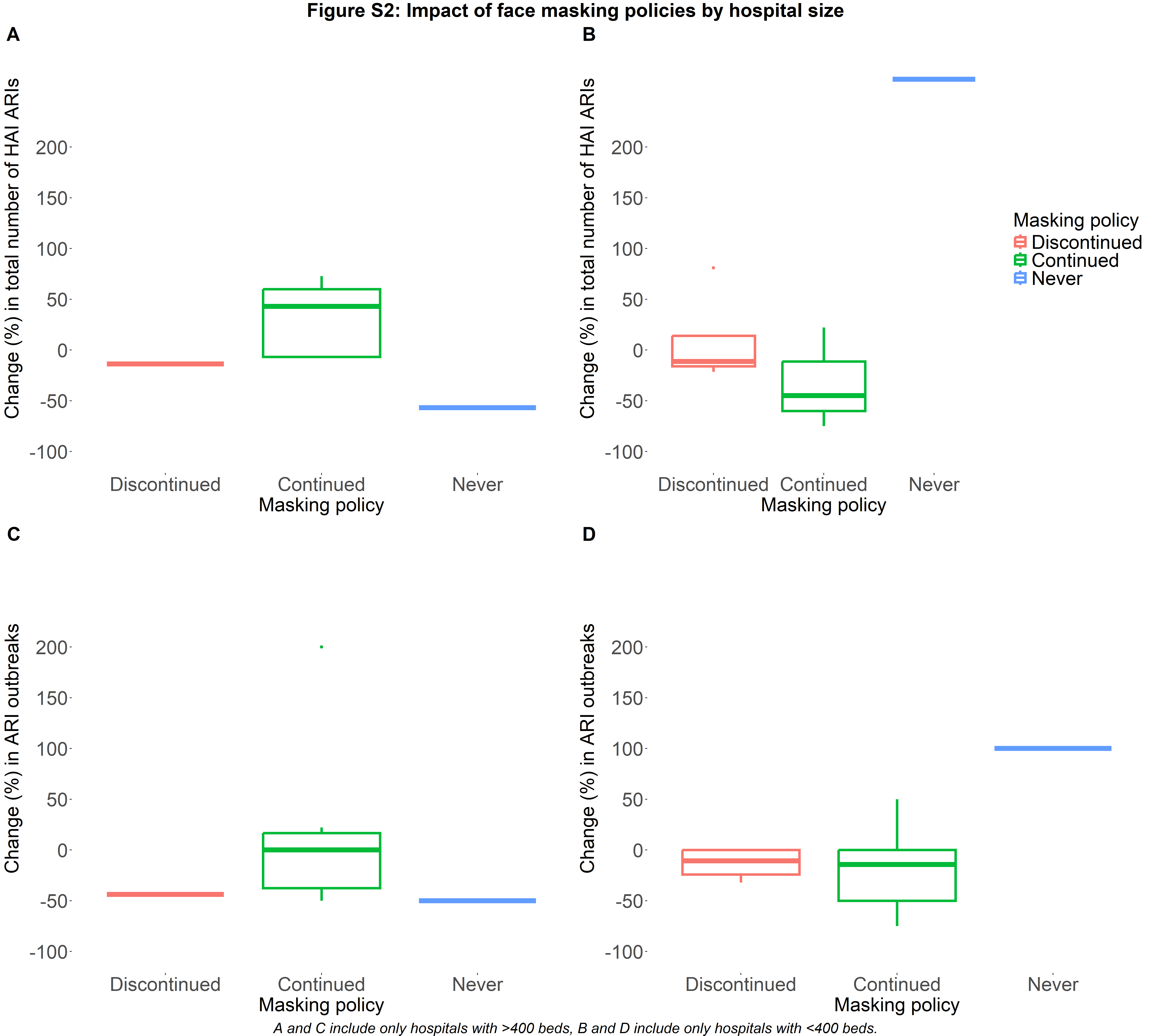

Supplement: Scheier et al. supplementary material 3 — Scheier et al. supplementary material [file S2732494X26104239sup003.zip › FigureS2.png]
