# Supplementary material for: Impact of masking policy on healthcare-associated acute respiratory infections in 18 hospitals in Southern Ontario
Source: Antimicrob Steward Healthc Epidemiol. 2026 Jun 1;6(1):e160. doi: 10.1017/ash.2026.10423 (PMC13227126; doi:10.1017/ash.2026.10423)
Supplement: Scheier et al. supplementary material 4 — Scheier et al. supplementary material [file S2732494X26104239sup004.pdf]

Supplement Table S1: Characteristics of sites

			Year 1						Year 2					
			Outbreaks			HA-Cases			Outbreaks			HA-Cases		
Site	Beds	Policy group	Covid	Influenza	RSV	Covid	Influenza	RSV	Covid	Influenza	RSV	Covid	Influenza	RSV
1	>400	Discontinued	19	8	1	298	55	25	13	3	3	203	61	60
2	<400	Continued	0	0	0	0	0	0	0	0	0	0	0	0
3	<400	Never	1	0	0	9	0	0	2	0	0	29	4	0
4	<400	Continued	2	0	0	n.r.	n.r.	n.r.	1	0	0	n.r.	n.r.	n.r.
5	>400	Discontinued	12	4	0	68	12	0	7	2	0	62	7	0
6	<400	Continued	2	0	0	n.r.	n.r.	n.r.	2	1	0	n.r.	n.r.	n.r.
7	<400	Continued	1	0	1	8	0	3	2	0	0	19	0	0
8	>400	Continued	12	1	1	87	6	2	11	2	1	78	8	2
9	<400	Discontinued	9	5	0	73	45	3	5	5	1	62	41	8
10	>400	Continued	14	0	0	136	9	17	8	3	1	128	51	19
11	<400	Continued	1	0	0	14	0	0	1	1	1	16	2	2
12	>400	Continued	8	1	0	113	26	8	6	5	0	76	45	16
13	<400	Discontinued	1	0	0	20	1	0	1	0	0	16	22	0
14	>400	Never	23	3	0	213	16	0	6	6	1	61	34	4
15	<400	Discontinued	2	0	0	14	0	0	2	0	0	11	0	0
16	<400	Continued	4	0	0	20	0	0	1	0	0	5	0	0
17	>400	Continued	4	0	0	40	3	2	0	2	0	46	20	6
18	<400	Continued	2	0	0	18	2	0	0	1	0	3	7	1
